# Antipsychotic Medication Adherence Among Outpatients Attending the Centre Neuro Psycho Pathologique in Kinshasa: A Cross-Sectional Pilot Study of Treatment Attitudes, Insight, and Side-Effect Burden

**DOI:** 10.7759/cureus.105916

**Published:** 2026-03-26

**Authors:** Yves Tshangala Kavunga, Julienne Nzuzi Mananga, Jael Won Vuvu Ngimbi, Philippe Ntalaja Kabuayi

**Affiliations:** 1 Psychiatry, Université Protestante au Congo, Kinshasa, COD; 2 Psychiatry, Centre Neuro Psycho Pathologique (CNPP), Kinshasa, COD; 3 General Medicine, University of Kinshasa, Kinshasa, COD; 4 Psychiatry, Dr Joseph Guislain Neuropsychiatric Centre of Lubumbashi, Brothers of Charity (CNPJG), Lubumbashi, COD

**Keywords:** adverse effects, antipsychotics, democratic republic of the congo, insight, kinshasa, medication adherence, outpatients, treatment attitude

## Abstract

Background and objective

Incomplete adherence to antipsychotics commonly undermines relapse prevention and functional stabilization in severe mental disorders. Published data from the Democratic Republic of the Congo (DRC) are scarce. The objective of this pilot study was to estimate antipsychotic medication adherence among outpatients attending the Centre Neuro Psycho Pathologique (CNPP) in Kinshasa, DRC, and examine associations with treatment attitude, insight, and adverse effects using brief standardized instruments.

Methods

We conducted a cross-sectional pilot study from April to May 2025 at the outpatient service of the CNPP in Kinshasa, DRC. Adult patients (≥18 years) who had been prescribed at least one antipsychotic for a minimum duration of five weeks were included. Adherence was measured with the 10-item Medication Adherence Rating Scale (MARS). Adherence was dichotomized as good adherence (MARS ≥ 7) and poor adherence (MARS < 7). The Drug Attitude Inventory (DAI) was used to assess treatment attitude. Insight was assessed with the 8-item Birchwood Insight Scale (BIS-8). The Glasgow Antipsychotic Side-Effect Scale (GASS) measured the adverse effects of antipsychotics. Correlations between scales were examined by Spearman’s rank correlation coefficient rho (ρ). Modelled associations were examined using logistic regression (exploratory, parsimonious models). Associations were explored using logistic regression. Significance was set at α = 0.05 with 95% confidence intervals (CI).

Results

A total of 62 outpatients were included: mean age 37.5 years, standard deviation (SD) 14.4; 37 (59.7%) were men. Poor adherence occurred in 28 patients (45.2%; 95% CI 33.4-57.5). Mean scores were 5.89 (SD 3.04) for MARS, 2.52 (SD 5.37) for DAI, 5.80 (SD 2.86) for BIS, and 20.63 (SD 20.84) for GASS. Of the enrolled patients, 39 (62.9%) had a positive treatment attitude, nine (14.5%) had good insight, and 12 (19.4%) had a severe side-effect burden. The MARS correlated with DAI (ρ=0.787; p<0.001) and BIS-8 (ρ=0.616; p<0.001) and inversely with GASS (ρ=-0.660; p<0.001). In the multivariable extended model, higher DAI (adjusted odds ratio (aOR) 0.54 per 1-point increase; 95% CI 0.32-0.93; p=0.025) and higher BIS-8 (aOR 0.39 per 1-point increase; 95% CI 0.17-0.87; p=0.022) were independently associated with lower odds of poor adherence; GASS was not independently associated (aOR 1.30; 95% CI 0.96-1.78; p=0.092).

Conclusion

Nearly half of the outpatients receiving antipsychotics in this Kinshasa pilot study reported poor adherence. Treatment attitude and insight, both modifiable, showed independent associations with poor adherence, while adverse effects were strongly correlated with adherence but were not independently associated after adjustment. These exploratory findings suggest that structured side-effect monitoring and shared decision-making may be relevant components to evaluate in future adherence-oriented interventions in constrained settings.

## Introduction

Mental disorders rank among the most disabling health conditions globally due to their substantial and enduring contribution to years lived with disability across regions and income settings [1]. Antipsychotics are commonly used to treat mental disorders, on or off-label [2]. Antipsychotic maintenance treatment is an important factor to maintain stability in conditions like schizophrenia or bipolar disorders and to prevent relapse [3]. However, the clinical benefits noted in controlled settings are often lowered in standard care due to incomplete adherence, which is frequent and frequently hidden [4,5].

Poor adherence to antipsychotics is associated with worse outcomes like symptom exacerbations, relapse, emergency presentations, and rehospitalization [4]. It also contributes to more use of health care and higher costs [4-7]. Studies have shown that poor adherence is associated with factors such as poor insight, negative beliefs or attitudes toward medication, adverse effects, substance use, and service-related barriers [6,8]. These associated factors are important in low- and middle-income contexts, where limited medication options, out-of-pocket costs, and limited follow-up resources can lead to treatment discontinuation [9,10]. In sub-Saharan Africa, a meta-analysis estimated that approximately half of patients with schizophrenia were nonadherent to antipsychotic medication, with treatment-related adverse effects and negative attitudes toward medication among the repeatedly implicated correlates [10].

Three modifiable, patient-centered constructs warrant focused attention. First is treatment attitude, which summarizes the subjective appraisal of benefits, perceived necessity, and acceptability of antipsychotic treatment and has long been linked to medication-taking behavior [11]. Second is insight; it relates to illness awareness and attribution of symptoms and is repeatedly associated with adherence patterns in schizophrenia [6,12]. Third is the adverse effects that constitute a proximal and actionable barrier. Systematic elicitation of side effects improves clinical detection and may inform dose optimization and shared decision-making [13].

In the Democratic Republic of the Congo (DRC), available evidence highlights a large treatment gap and limited formal supply of mental health services in several settings, emphasizing the need for locally generated, pragmatic data to inform service-level interventions [10]. Against this background, the objective of this pilot study was to estimate antipsychotic medication adherence among outpatients attending the Centre Neuro Psycho Pathologique (CNPP) in Kinshasa and examine associations with treatment attitude, insight, and adverse effects using brief standardized instruments.

## Materials and methods

Study design and setting

This cross-sectional pilot study was conducted at the outpatient service of the CNPP at the University of Kinshasa (Kinshasa, DRC). The protocol, including the data from this study, was submitted and approved by the Ethics Committee of the School of Public Health, University of Kinshasa (approval number: ESP/CE/21/25). All participants provided informed consent prior to inclusion. The study was designed and reported in accordance with the Strengthening the Reporting of Observational Studies in Epidemiology (STROBE) guidelines (see Appendix A) for cross-sectional studies [14]. 

Data were collected during routine ambulatory follow-up from April 1st to May 1st, 2025. The study was designed as a pilot to generate the first local estimates of antipsychotic adherence in this setting, quantify the distribution of key patient-reported determinants (treatment attitudes, insight, and side effect burden), and assess the feasibility of standardized data collection in a tertiary hospital. We used a pragmatic sample size constrained by the recruitment period. No formal sample-size calculation was made a priori because the primary objective was to assess the feasibility of standardized data collection and to generate preliminary estimates for future hypothesis-driven research.

The CNPP is the main public neuropsychiatric facility affiliated with the University of Kinshasa and is a tertiary referral center for the city-province of Kinshasa (estimated population ≥ 17 million) [15] and referred patients from other provinces of the DRC [16]. The outpatient services provide follow-up for patients discharged from inpatient services and those referred from primary and secondary facilities [17]. Because CNPP is a tertiary hospital, the case mix may be more clinically complex than in community or primary care settings. Therefore, the generalizability of the prevalence estimate and associated factors should be interpreted with caution.

Participants

Eligible participants were adults (≥ 18 years) followed in ambulatory care at CNPP and had been on an unchanged antipsychotic regimen for at least five weeks at the time of the survey. This threshold was chosen to ensure that participants had attained pharmacokinetic steady state for the majority of oral antipsychotics and that early-onset extrapyramidal and metabolic side effects would have manifested themselves. This lessens confounding from recent dose changes or transient adverse effects. Patients who could not complete the questionnaires (e.g., severe cognitive impairment, marked behavioral disorganization, or acute agitation at the visit) were excluded from the study. The sample was a pragmatic convenience series of consecutive eligible patients encountered during the study period. Data was collected by a senior psychiatrist assistant through a face-to-face interview. Recruitment took place on all consultation days during the study period. Each consecutive eligible participant was systematically approached. No formal screening log was maintained, but reasons for exclusion were documented (see Appendix B for participant exclusion criteria). The total number of patients attending the outpatient service during the recruitment period was not recorded.

Data sources and variables

Clinical and treatment-related information was obtained from the patient interview and medical records, including age, gender, marital status, education, income, activity, accompaniment to the visit, duration of the trouble, and DSM-5 diagnosis recorded in the file by the psychiatrist in charge as part of routine clinical assessment. No structured diagnostic interview was administered. Diagnostic inter-rater reliability was not formally evaluated for this pilot survey. This is a methodological limitation. Diagnoses were grouped for analytic purposes into the following categories: schizophrenia-spectrum and primary psychotic disorders (including schizophrenia, schizoaffective disorder, schizophreniform disorder, brief psychotic disorder, and chronic hallucinatory psychosis), substance-induced psychotic disorder, bipolar disorder, depressive disorder, neurocognitive disorder, and other.

Current treatment was recorded (molecule and dose). Antipsychotic polypharmacy was defined as the concurrent use of ≥2 antipsychotics. Exposure to long-acting injectable (LAI) haloperidol decanoate (the only LAI used) was coded present if listed among current medications. The total antipsychotic daily dose was expressed as chlorpromazine equivalent (EqCPZ) based on published conversion tables and summed when more than one was prescribed [18-21]. The prescribed daily dose was converted using the molecule-specific EqCPZ ratio (e.g., chlorpromazine 100 mg=100 mg EqCPZ; haloperidol 2 mg=100 mg EqCPZ; risperidone 2 mg=100 mg EqCPZ; olanzapine 5 mg=100 mg EqCPZ). For haloperidol decanoate, the administered dose and injection interval were converted to an average daily dose before applying the EqCPZ ratio [20]. The EqCPZ was analyzed per 100 mg increase (EqCPZ/100) to improve interpretability. These conversion ratios covered all antipsychotic molecules prescribed in the study sample (see Appendix C).

Measures

All questionnaires were administered in French. For MARS, a previously published French version was used. For the DAI-10, BIS-8, and GASS, French-language versions/translations of the original instruments were used for study administration in our setting. Each participant read and completed questionnaires independently. The interviewer was available to clarify any ambiguous items at the participant's request. The average completion time for the full survey was approximately 15 to 20 minutes. Prior to the study, the survey package was pre-tested in five outpatients to ensure comprehension and feasibility within routine visits. This pre-test served as a feasibility check for item comprehension and administration logistics rather than a formal cultural adaptation procedure. No wording changes were required.

Medication adherence

Medication adherence was measured with the 10-item Medication Adherence Rating Scale (MARS). The total score ranges from 0 to 10. Higher scores indicate better adherence [22,23]. Adherence was dichotomized as good adherence (MARS total ≥ 7) versus poor adherence (MARS < 7) [22,24]. We used the French translation of the MARS validation [25].

Treatment attitude

We assessed treatment attitude toward antipsychotics with the 10-item Drug Attitude Inventory (DAI). Items were scored +1/-1 and summed to a total score ranging from -10 to +10. Higher scores indicate a more favorable attitude toward medication [11]. The DAI was categorized as negative (<0), neutral (=0), or positive (>0). The questionnaire was administered using the French translation. No local validation study has been conducted in the DRC.

Insight

Insight was assessed with the 8-item Birchwood Insight Scale (BIS-8). Total score ranges from 0 to 12. Higher scores indicate better insight [12]. We used a ≥ 9 threshold to describe 'good insight' vs. 'poor insight' [26,27]. The BIS-8 was administered using the French translation. The DRC-specific validation data are unavailable.

Adverse effects were assessed using the Glasgow Antipsychotic Side-Effect Scale (GASS). It is a patient-rated checklist scored 0-3 per item and summed to a total score [13]. Side-effect severity was categorized as absent/mild (0-21), moderate (22-42), and severe (≥ 43) [28]. The GASS was administered using the French-translated version of the original English-language scale. Interpretation of absolute scores should consider the absence of local cultural adaptation data.

Statistical analysis

Analyses were performed using SPSS Statistics version 31 (IBM Corp., Armonk, NY, USA). All tests were two-sided with α = 0.05. We used the Shapiro-Wilk test [29] to check distributional assumptions. Continuous variables were summarized as mean ± standard deviation (SD) when normally distributed and as median (interquartile range, IQR) when skewed. Means were additionally provided for key psychometric totals to facilitate comparison with the literature. The prevalence of poor adherence was reported with 95% confidence intervals (CI).

Internal consistency of psychometric scales (MARS, DAI, BIS, and GASS) was evaluated using Cronbach’s alpha, acknowledging the tau-equivalence assumption. Corrected item-total correlations and alpha-if-item-deleted statistics were examined. We evaluated associations between continuous scale scores using Spearman’s rank correlation rho (ρ). For bivariable comparisons (good vs. poor adherence), categorical variables were compared using Pearson’s chi-square test or Fisher’s exact test when expected cell counts were small. Continuous variables were compared using Student’s t-test or the Mann-Whitney U test as appropriate.

We used univariable logistic regression to estimate crude odds ratios (ORs) for poor adherence. Multivariable logistic regression was conducted with parsimonious models to limit sparse-data bias, given the sample size. We specified a priori a 'clinical/treatment' model and included gender, accompaniment, antipsychotic type, polypharmacy, EqCPZ (per +100 mg), and haloperidol decanoate exposure (events per variable (EPV) = 4.0). The extended model added the psychometric predictors (DAI, BIS, and GASS total scores) with EPV = 2.8. Multicollinearity was assessed using variance inflation factors (VIF) (see Appendix D). Model fit and performance were summarized using the likelihood-ratio chi-square test, the Hosmer-Lemeshow goodness-of-fit test [30], and the area under the receiver operating characteristic curve (AUC). The AUC is reported as the apparent (in-sample) value and as an optimism-corrected estimate based on 500 bootstrap resamples. Missing values were handled using complete-case analysis for each model. Key psychometric measures and the adherence outcome had no missing data. No missing data were observed for sociodemographic, clinical, or treatment variables. Sensitivity analysis using Firth’s penalized (bias-reduced) logistic regression for the clinical/treatment and extended models was performed, given sparse cells and quasi-complete separation for some predictors.

## Results

Participants and baseline characteristics

Table 1 shows the participants’ sociodemographic, clinical, and antipsychotic treatment characteristics. A total of 62 outpatients receiving antipsychotic treatment were included. The mean age was 37.5 ± 14.4 years; 37 participants were male (59.7%), and 40 patients (64.5%) who attended the visit were accompanied. Participant diagnoses were schizophrenia-spectrum/psychotic disorders in 42/62 (67.7%), mood disorders in 10/62 (16.1%), neurocognitive disorders in 4/62 (6.5%), and other diagnoses in 6/62 (9.7%). Of the included patients, 23/62 (37.1%) received atypical antipsychotics only, 28/62 (45.2%) received typical antipsychotics only, and 11/62 (17.7%) received a combination of typical and atypical antipsychotics; 16 patients (25.8%) had polypharmacy. Haloperidol decanoate was prescribed to 10 patients (16.1%). The median total antipsychotic exposure was 233.5 mg EqCPZ per day (IQR 100.0-500.0). The median duration of the disorder was 3.0 years (IQR 1.0-8.0), and the median of prior hospitalizations was 1.0 (IQR 0.0-1.0).

**Table 1:** Participant sociodemographic, clinical and antipsychotic treatment characteristics The Shapiro-Wilk test for continuous variables was applied. SD: Standard deviation; IQR: Interquartile range; EqCPZ: Chlorpromazine equivalents (mg/day)

Variable	Total (n=62)	W (Shapiro-Wilk)	p (Shapiro-wilk)
Age, mean ± SD (years)	37.5 ± 14.4	0.909618	0.0002374649
Gender male, n(%)	37 (59.7)	-	-
Married, n(%)	19 (30.6)	-	-
Years of study, median (IQR)	13.0 (12.0-15.0)	0.903025	0.0001328778
Income-generating activity (yes), n(%)	13 (21.0)	-	-
Diagnosis group: schizophrenia-spectrum / primary psychotic, n(%)	42 (67.7)	-	-
Diagnosis group: substance-induced psychotic disorder, n(%)	5 (8.1)	-	-
Diagnosis group: bipolar disorder, n(%)	6 (9.7)	-	-
Diagnosis group: depressive disorder, n(%)	4 (6.5)	-	-
Diagnosis group: neurocognitive disorder, n(%)	4 (6.5)	-	-
Diagnosis group: other, n(%)	1 (1.6)	-	-
Accompaniment, n(%)	40 (64.5)	-	-
Antipsychotic type: atypical only, n(%)	23 (37.1)	-	-
Antipsychotic type: typical only, n(%)	28 (45.2)	-	-
Antipsychotic type: combination, n(%)	11 (17.7)	-	-
Antipsychotic polypharmacy (≥2), n(%)	16 (25.8)	-	-
Number of antipsychotics, median (IQR)	1.0 (1.0-1.8)	0.580401	4.880485x10^-12^
Total number of medications, median (IQR)	3.0 (2.0-3.0)	0.909678	0.0002387270
Haloperidol decanoate, n (%)	10 (16.1)	-	-
EqCPZ, median (IQR) mg/day	233.5 (100.0-500.0)	0.876400	0.0000151979
Previous hospitalizations, median (IQR)	1.0 (0.0-1.0)	0.826248	4.648403x10^-7^
Duration of disorder, median (IQR) years	3.0 (1.0-8.0)	0.795280	7.196232x10^-8^

Adherence and psychometric measures

The mean MARS score was 5.89 ± 3.04 (median 7.0; IQR 3.25-8.0). Poor adherence was observed in 28/62 patients (45.2%; 95% CI 33.4-57.5). Overall mean scores were 2.52 ± 5.37 for DAI, 5.80 ± 2.86 for BIS, and 20.63 ± 20.84 for GASS. A positive treatment attitude was present in 39/62 patients (62.9%), neutral in eight/62 (12.9%), and negative in 15/62 (24.2%). Good insight was identified in nine/62 patients (14.5 %). Side-effect burden was classified as absent/mild in 47/62 (75.8%), moderate in three/62 (4.8%), and severe in 12/62 (19.4%). Table 2 presents adherence outcomes and psychometric scores. Internal consistency was acceptable to excellent; Cronbach’s α = 0.830 (MARS), 0.758 (DAI), 0.730 (BIS), and 0.963 (GASS). See Table 3 for correlations across scales and reliability coefficients.

**Table 2:** Medication adherence and psychometric measures The BIS was the only scale not deviating significantly from normality. MARS: Medication Adherence Rating Scale, DAI: Drug Attitude Inventory, BIS: Birchwood Insight Scale, GASS: Glasgow Antipsychotic Side-effect Scale, CI: Confidence interval, SD: Standard deviation, IQR: Interquartile range

Measure	Total (n=62)	W (Shapiro-Wilk)	p (Shapiro-Wilk)
MARS, Mean ± SD	5.89 ± 3.04		
MARS, median (IQR)	7.00(3.25-8.00)	0.899140	0.0000952111
Poor adherence, n/n (%) CI95%	28/62 (45.2) 33.4-57.5		
DAI, mean ± SD	2.52 ± 5.37	0.934995	0.0026947357
DAI positive attitude, n(%)	39 (62.9)		
DAI neutral attitude, n(%)	8 (12.9)		
DAI negative attitude, n(%)	15 (24.2)		
BIS, mean ± SD	5.80 ± 2.86	0.964524	0.0701181902
BIS good insight, n(%)	9 (14.5)		
GASS, mean ± SD	20.63 ± 20.84	0.744034	4.661350x10^-9^
GASS absent/mild, n(%)	47 (75.8)		
GASS moderate, n(%)	3 (4.8)		
GASS severe, n(%)	12 (19.4)		

**Table 3:** Correlations between adherence, treatment attitude, insight, and side effects, and internal consistency of scales Off-diagonal cells are Spearman’s correlations and the exact two-sided p-value. Cronbach’s alpha values are reported in the sixth column. All correlations are significant at p<0.05. The complete table is provided in Appendix E. MARS: Medication Adherence Rating Scale, DAI: Drug Attitude Inventory, BIS: Birchwood Insight Scale, GASS: Glasgow Antipsychotic Side-effect Scale

Scale	MARS ρ (p)	DAI	BIS	GASS	Cronbach α
MARS	-	0.787 (p=0.000000000000033)	0.616 (p=0.000000098260369)	-0.660 (p=0.000000005291995)	0.830
DAI	0.787 (p=0.000000000000033)	-	0.481 (p=0.000076313358278)	-0.570 (p=0.000001319544528)	0.758
BIS	0.616 (p=0.000000098260369)	0.481 (p=0.000076313358278)	-	-0.353 (p=0.004884424173122)	0.730
GASS	-0.660 (p=0.000000005291995)	-0.570 (p=0.000001319544528)	-0.353 (p=0.004884424173122)	-	0.963

We examined the item-total statistics for each of the four scales. For MARS (α = 0.830), the corrected item-total correlations ranged from 0.345 (item 6) to 0.707 (item 4), while the alpha-if-item-deleted values ranged from 0.796 to 0.832. Removing any one item would not appreciably raise the overall alpha. The corrected item-total correlations for DAI (α = 0.758) ranged from 0.245 (item 10) to 0.579 (item 9), and the alpha-if-item-deleted values ranged from 0.715 to 0.763. The corrected item-total correlations for BIS (α = 0.730) were between 0.136 (item 2) and 0.663 (item 6), and the alpha-if-item-deleted values were between 0.648 and 0.752. If item 2 ('my doctor is right in prescribing medication for me') were taken out, alpha would increase up to 0.752. The item was maintained, nevertheless, to ensure the content was valid and could be compared to past BIS investigations.

It's important to note that the GASS has a very high alpha (α = 0.963, 23 items). The corrected item-total correlations ranged from -0.094 (item 21, which questions about sexual function) to 0.924 (item 18). Twenty-two out of twenty-three items had adjusted item-total correlations of more than 0.40, indicating a strong association among the items. Item 21 had a negative adjusted item-total correlation (r = -0.094), and removing it would raise alpha to 0.969.

Bivariable comparisons by adherence status

Table 4 compares clinical and treatment factors by adherence status. Compared with adherent patients, those with poor adherence were more frequently men (22/28 vs. 15/34; p = 0.006) and more often accompanied to the visit (22/28 vs. 18/34; p = 0.036). They had higher antipsychotic dose exposure (EqCPZ median 500.0 vs 175.0; p = 0.005). Haloperidol decanoate was less common among poorly adherent patients, but not significant (Fisher’s p = 0.097). Polypharmacy was not associated with adherence status (p = 0.301).

**Table 4:** Bivariable comparisons by adherence status (good vs. poor adherence) Data Percentages are column percentages and may not sum to 100 due to rounding. Good adherence was defined as a MARS total score ≥7; poor adherence as MARS <7. EqCPZ: Chlorpromazine equivalents (mg/day), DAI: Drug Attitude Inventory, BIS: Birchwood Insight Scale, GASS: Glasgow Antipsychotic Side-effect Scale, MARS: Medication Adherence Rating Scale, df: Degrees of freedom, SD: Standard deviation, IQR: Interquartile range

Factor	Good adherence (n=34)	Poor adherence (n=28)	Test (df)	p-value
Gender male, n (%)	15 (44.1)	22 (78.6)	χ²(1)=7.57	0.006
Married, n (%)	12 (35.3)	7 (25.0)	χ²(1)=0.77	0.382
Years of study, median (IQR)	15.0 (12.0-15.0)	12.0 (12.0-15.2)	U=521	0.517
Income-generating activity (yes), n (%)	6 (17.6)	7 (25.0)	χ²(1)=0.50	0.479
Diagnosis group, n (%)			χ²(5)=7.05	0.217
- Schizophrenia-spectrum/primary psychotic	22 (64.7)	20 (71.4)		
- Substance-induced psychotic disorder	4 (11.8)	1 (3.6)		
- Bipolar disorder	2 (5.9)	4 (14.3)		
- Depressive disorder	2 (5.9)	2 (7.1)		
- Neurocognitive disorder	4 (11.8)	0 (0.0)		
- Other	0 (0.0)	1 (3.6)		
Accompaniment (yes), n (%)	18 (52.9)	22 (78.6)	χ²(1)=4.41	0.036
Antipsychotic type, n (%)			χ²(2)=3.64	0.162
- Atypical only	16 (47.1)	7 (25.0)		
- Typical only	12 (35.3)	16 (57.1)		
- Combination (typical + atypical)	6 (17.6)	5 (17.9)		
Antipsychotic polypharmacy (≥2), n(%)	7 (20.6)	9 (32.1)	χ²(1)=1.07	0.301
Number of antipsychotics, median (IQR)	1.0 (1.0-1.0)	1.0 (1.0-2.0)	U=422	0.320
Total number of medications, median (IQR)	2.5 (2.0-3.0)	3.0 (2.0-4.0)	U=347	0.059
Haloperidol decanoate, n (%)	8 (23.5)	2 (7.1)	Fisher's exact	0.097
EqCPZ, median (IQR) mg/day	175.0 (100.0-342.0)	500.0 (175.0-525.0)	U=279	0.005
Previous hospitalizations, median (IQR)	1.0 (0.0-1.0)	1.0 (1.0-2.0)	U=407	0.297
Duration of disorder (years), median (IQR)	3.0 (1.0-8.0)	3.5 (1.0-9.0)	U=467	0.904
DAI, mean ± SD	5.88 ± 3.07	-1.57 ± 4.69	U=859.5	0.000000048263269
BIS, mean ± SD	7.29 ± 2.19	3.98 ± 2.53	U=777	0.000020274546116
GASS, mean ± SD	9.68 ± 6.03	33.93 ± 24.53	U=165.5	0.000011293272583
DAI negative attitude, n (%)	0 (0.0)	15 (53.6)	Fisher's exact	0.000000402375642
GASS moderate/severe, n (%)	2 (5.9)	13 (46.4)	Fisher's exact	0.000260738407633

Psychometric differences were marked. Poor adherent patients had lower DAI scores (-1.57 ± 4.69 vs. 5.88 ± 3.07; p < 0.001), lower BIS scores (3.98 ± 2.53 vs. 7.29 ± 2.19; p < 0.001), and higher GASS scores (33.93 ± 24.53 vs. 9.68 ± 6.03; p < 0.001). All participants with a negative treatment attitude were in the poor adherence group (15/15). Severe adverse effects occurred exclusively in the poor adherence group (12/12). When moderate and severe categories were combined, poor adherence was present in 13/15 vs. 15/47 in the absent/mild group (Fisher’s p < 0.001).

Correlations between adherence, attitude, insight, and adverse effects

The MARS total score correlated strongly with DAI (Spearman ρ = 0.787; p < 0.001) and moderately with BIS (ρ = 0.616; p < 0.001). The MARS correlated inversely with GASS (ρ = -0.660; p < 0.001). The DAI correlated positively with BIS (ρ = 0.481; p < 0.001) and negatively with GASS (ρ = -0.570; p < 0.001). The BIS also correlated negatively with GASS (ρ = -0.353; p = 0.005).

Logistic regression analyses for poor adherence (MARS < 7)

Table 5 presents univariable and multivariable logistic regression analyses of factors associated with poor adherence. In univariable logistic regression, poor adherence was associated with male gender (OR 4.64; 95% CI 1.50-14.35; p = 0.008), being accompanied (OR 3.26; 95% CI 1.06-10.05; p = 0.040), higher EqCPZ (per 100 mg: OR 1.34; 95% CI 1.06-1.70; p = 0.015), lower BIS (per 1-point increase: OR 0.57; 95% CI 0.43-0.76; p < 0.001), and higher GASS (per 1-point increase: OR 1.12; 95% CI 1.03-1.22; p = 0.007).

**Table 5:** Logistic regression analyses for poor adherence (MARS < 7) The clinical/treatment model included gender, accompaniment, antipsychotic type (reference: atypical only), polypharmacy, EqCPZ (per +100 mg/day), and haloperidol decanoate EPV ≈4.0). Model fit: likelihood-ratio χ²(7)=25.07 (p=0.00074); Hosmer-Lemeshow χ²(8)=7.72 (p=0.461). Discrimination: apparent AUC=0.842 (95% bootstrap CI 0.733-0.935); optimism-corrected AUC=0.770 (bootstrap, 500 resamples). Multicollinearity was low (max VIF=2.75). The extended model added DAI, BIS, and GASS totals (EPV ≈2.8). Model fit: likelihood-ratio χ²(10)=56.20 (p<0.001); Hosmer-Lemeshow χ²(8)=5.65 (p=0.687). Discrimination: apparent AUC=0.962 (95% bootstrap CI 0.910-0.998); optimism-corrected AUC=0.911 (bootstrap, 500 resamples). Multicollinearity remained acceptable (max VIF=2.94). OR: Odds ratio, aO: Adjusted odds ratio, CI: Confidence interval, EqCPZ: Chlorpromazine equivalents (mg/day), EPV: Events per variable, AUC: Area under the receiver operating characteristic curve, VIF: Variance inflation factors, df: Degrees of freedom, DAI: Drug Attitude Inventory, BIS: Birchwood Insight Scale, GASS: Glasgow Antipsychotic Side-effect Scale, MARS: Medication Adherence Rating Scale

Factor	OR (95% CI)	Wald χ² (df = 1)	p-value	aOR (clinical/treatment)	Wald χ²(df = 1)	p-value	aOR (extended)	Wald χ²(df) = 1	p-value
Gender, male	4.64 (1.50-14.35)	7.12	0.008	4.85 (1.20-19.56)	4.93	0.026	3.97 (0.39-40.16)	1.36	0.243
Accompaniment (yes)	3.26 (1.06-10.05)	4.23	0.040	2.79 (0.64-12.09)	1.89	0.170	0.24 (0.01-3.94)	1.00	0.318
Total medications (per +1)	1.53 (0.97-2.42)	3.42	0.064	-	-	-	-	-	-
Total antipsychotics (per +1)	1.57 (0.59-4.16)	0.83	0.364	-	-	-	-	-	-
Antipsychotic polypharmacy (>=2)	1.83 (0.58-5.76)	1.06	0.304	0.30 (0.03-2.55)	1.22	0.269	0.28 (<0.01-17.87)	0.36	0.548
Antipsychotic type: Typical only (ref atypical)	3.05 (0.95-9.74)	3.54	0.060	2.34 (0.39-14.06)	0.87	0.351	3.77 (0.22-65.00)	0.84	0.361
Antipsychotic type: Combination (ref atypical)	1.90 (0.43-8.39)	0.73	0.394	6.53 (0.38-112.93)	1.67	0.197	0.07 (<0.01-65.50)	0.59	0.444
EqCPZ (per +100 mg)	1.34 (1.06-1.70)	5.89	0.015	1.42 (1.00-2.02)	3.85	0.050	0.87 (0.50-1.51)	0.25	0.614
Haloperidol decanoate	0.25 (0.05-1.29)	2.74	0.098	0.03 (0.01-0.47)	6.25	0.012	0.68 (0.01-39.45)	0.03	0.854
DAI (per 1-point increase)	0.63 (0.51-0.79)	16.86	4.0276e-05	-	-	-	0.54 (0.32-0.93)	5.00	0.025
BIS (per 1-point increase)	0.57 (0.43-0.76)	15.36	8.8914e-05	-	-	-	0.39 (0.17-0.87)	5.29	0.022
GASS (per 1-point increase)	1.12 (1.03-1.22)	7.29	0.006943	-	-	-	1.30 (0.96-1.78)	2.84	0.092

In the multivariable clinical/treatment model, male gender (adjusted odds ratio (aOR) 4.85 (1.20-19.56); p = 0.026) remained independently associated with poor adherence. Haloperidol decanoate (aOR 0.03 (<0.01-0.47); p = 0.012) became significant, while EqCPZ showed a borderline association (aOR 1.42 (1.00-2.02); p = 0.050). In the extended model adding DAI, BIS, and GASS, both DAI (aOR 0.54 (0.32-0.93); p = 0.025) and BIS (aOR 0.39 (0.17-0.87); p = 0.022) remained independently associated with poor adherence. GASS showed a non-significant association (aOR 1.30 (0.96-1.78); p = 0.092).

Sensitivity analysis using Firth’s penalized logistic regression yielded similar conclusions for the psychometric measures (see Appendix F). For DAI (per +1 point), the Firth's OR was 0.65 (95% CI 0.53-0.80; p<0.001). For BIS (per +1 point), the Firth's OR was 0.59 (95% CI 0.45-0.77; p<0.001). For GASS (per +1 point), the Firth's OR was 1.10 (95% CI 1.03-1.17; p=0.004). These estimates were slightly less than the null, but the direction and inference were unchanged. In the Firth clinical/treatment model, the haloperidol decanoate estimate was attenuated (Firth's aOR 0.07; 95% CI 0.001-0.72; p = 0.026) in comparison to the standard maximum-likelihood estimate (aOR 0.03). This supports a cautious interpretation of this association.

## Discussion

In this study, nearly one in two participants met criteria for poor antipsychotic adherence using the MARS cut-off (MARS < 7). This magnitude is broadly consistent with the persistent adherence gap described in schizophrenia-spectrum disorders across settings, where nonadherence is common and clinically consequential despite the established efficacy of antipsychotics for relapse prevention [3-6,8]. Given that adherence was measured by self-report, the observed prevalence should be interpreted as a conservative estimate rather than an upper bound due to social desirability and recall effects of self-reporting [22,23].

Interpretation of the main associations

The most informative correlates clustered around modifiable, patient-centered dimensions: treatment attitude (DAI), insight (BIS), and adverse-effect burden (GASS). In the extended multivariable model, both DAI and BIS remained independently associated with adherence status. This pattern aligns with the conceptual view that adherence behaviors are strongly driven by subjective appraisal of medication (perceived benefits, acceptability, and necessity) and by illness-related awareness and attribution processes [6,8,11,12]. The DAI was developed to identify the experiential and attitudinal factors that influence daily medication-taking decisions. The results of this study support its clinical usefulness as a brief indicator for adherence vulnerability [11].

Insight showed that adherent patients reported elevated BIS scores. The BIS maintained an independent association after adjustment for clinical and treatment covariates. Previous research showed that impaired insight was associated with discontinuation and inconsistent use of antipsychotics, partially due to reduced perceived need for treatment and reduced engagement in follow-up care [6,8,12]. This supports focusing on interventions that improve shared decision-making and collaborative illness understanding, instead of just changing medications or controlling symptoms.

Adverse-effect burden (GASS) was higher in the poor-adherence group (see Appendix G). It showed a negative correlation with MARS. Side effects are frequently cited as a reason for skipping doses or discontinuing treatment [5,8]. They lower the quality of life, functioning, and the willingness to continue with treatment [5,6,8,13]. In the fully adjusted model, the GASS association became not significant. This may be consistent with a mediation model in the sense that adverse effects may indirectly affect adherence by influencing treatment attitude and acceptability, rather than functioning as an entirely independent variable. Cross-sectional data cannot resolve directionality. But the observed configuration is hypothetically compatible with a pathway in which adverse effects may be associated with worse medication attitudes and, thereby, lower adherence [5,11,13].

The association between being accompanied to the visit and poor adherence should be interpreted carefully. In routine ambulatory care, accompaniment may serve as an indicator of increased clinical severity, cognitive or functional impairment, or family concern arising from recent decompensation [31]. These factors could be upstream determinants of both accompaniment and nonadherence. Reverse causality is also possible: patients who are nonadherent and unstable may be more likely to be accompanied by relatives to facilitate access to care [32]. Additionally, a selection bias may operate: some patients who would otherwise have missed their appointment may only attend because they are accompanied, leading to an over-representation of poorly adherent patients among accompanied attendees. Because symptom severity and functional status were not measured, residual confounding cannot be excluded.

Dose, antipsychotic class, and long-acting formulations

Higher antipsychotic dose exposure (EqCPZ) was associated with poor adherence in univariable analyses and remained borderline in the clinical/treatment model, suggesting that dose burden may contribute to nonadherence. Antipsychotic type and polypharmacy showed no associations once clinical covariates and psychometric measures were considered. It is important to recognize that the EqCPZ has limitations because it compresses different pharmacodynamic profiles into a single metric [18,19]. Still, the association between higher EqCPZ and higher adverse-effect burden in poor-adherent patients supports a pragmatic implication: adherence-oriented care should focus on regular reviews of the minimum effective dose, simplification of treatment plans, and structured adverse-effect monitoring [13,19].

Exposure to haloperidol decanoate showed a protective association in the clinical model, with an extreme adjusted OR and a very wide CI, which may indicate instability. This pattern aligns with sparse-cell bias and potential quasi-separation in a small sample. In Firth penalized sensitivity analyses (Appendix F), the association’s direction was similar. The magnitude was less extreme. This supports cautious interpretation. The LAI antipsychotics can reduce the burden of daily pill-taking, provide clearer signals of treatment interruption, and improve continuity of taking medication. However, the effect varies depending on the design and context [3,5,6]. In this study, interpretation must be cautious. Depot use may be preferentially prescribed to patients perceived as high risk for poor adherence or, on the contrary, to those who are open to continuing treatment. The attenuation of the depot association in the extended model hypothesizes that attitude and insight may partially explain who receives and benefits from a long-acting formulation [11,12]. This finding is hypothesis-generating and should be interpreted cautiously, given the small number of participants receiving LAI haloperidol.

Limitations

Several limitations temper inference. First, the cross-sectional design precludes causal interpretation and does not establish temporal ordering between adverse effects, attitudes, insight, and adherence. Observed associations may reflect reverse causality or bidirectional relationships. Second, the sample was small, monocentric, and recruited by convenience in ambulatory care. This limits external validity. Third, adherence, attitude, insight, and adverse effects were self-reported and may have led to common-method bias and social desirability effects. A Harman single-factor exploration did not suggest the predominance of a single factor. But bias cannot be excluded [24]. Fourth, multivariable analyses were constrained by sparse data (28 outcome events), resulting in low EPV and unstable estimates for some predictors, including extensive CI and possible quasi-separation (particularly for haloperidol decanoate). The low EPV ratios (4.0 for the clinical/treatment model and 2.8 for the extended model) imply that parameter estimations should be seen as exploratory. This is shown by the very wide confidence intervals for several factors (e.g., haloperidol decanoate), which reflect instability. Moreover, some potentially significant confounders (such as symptom severity, substance use, therapeutic relationship, and treatment affordability) were not assessed and therefore could not be integrated into the current analysis. Finally, questionnaires were administered in French, and none have been formally validated in the DRC. Although the pre-test in five patients confirmed item comprehension, this does not substitute for a formal cross-cultural validation process. This should be considered when interpreting absolute scale values.

## Conclusions

In a care setting facing major structural constraints, adherence interventions need to be feasible, low-cost, and closely integrated into routine follow-up. Although the cross-sectional design of this pilot study precludes causal conclusions, the observed associations suggest a clinical approach that focuses on quick standardized screening and rapid corrective action. This includes systematically asking about side effects using a structured tool, talking to the patient about their medication beliefs to guide counseling, and using psychoeducation methods and engagement that are adapted to the patient’s explanatory model and family context. This method fits with the idea that a combination of strategies is better than just one-on-one counseling for dealing with the many factors that affect adherence. In addition, if possible, using LAI for patients who have frequent breaks may help keep exposure stable over time. Larger longitudinal studies that use objective adherence measures and take into account other important contextual factors like substance use, symptom severity, affordability and continuity of supply, transportation barriers, and therapeutic alliance are needed to examine these associations and then guide targeted interventions in the DRC.

## Appendices

Appendix A

The STROBE checklist is presented in Table 6.

**Table 6:** STROBE guidelines STROBE: Strengthening the reporting of observational studies in epidemiology [14]

Parameters		Item no.	Recommendation	Status
Title and abstract		1	(a) Indicate the study’s design with a commonly used term in the title or the abstract	Covered
			(b) Provide in the abstract an informative and balanced summary of what was done and what was found	Covered
Introduction	Background/rationale	2	Explain the scientific background and rationale for the investigation being reported	Covered
	Objectives	3	State-specific objectives, including any prespecified hypotheses	Covered
Methods	Study design	4	Present key elements of the study design early in the paper	Covered
	Setting	5	Describe the setting, locations, and relevant dates, including periods of recruitment, exposure, follow-up, and data collection	Covered
	Participants	6	(a) Give the eligibility criteria, and the sources and methods of selection of participants	Covered
	Variables	7	Clearly define all outcomes, exposures, predictors, potential confounders, and effect modifiers. Give diagnostic criteria, if applicable	Covered
	Data sources/ measurement	8*	For each variable of interest, give sources of data and details of methods of assessment (measurement). Describe the comparability of assessment methods if there is more than one group	Covered
	Bias	9	Describe any efforts to address potential sources of bias	Covered
	Study size	10	Explain how the study size was arrived at	Covered
	Quantitative variables	11	Explain how quantitative variables were handled in the analyses. If applicable, describe which groupings were chosen and why	Covered
	Statistical methods	12	(a) Describe all statistical methods, including those used to control for confounding	Covered
			(b) Describe any methods used to examine subgroups and interactions	Not applicable
			(c) Explain how missing data were addressed	Covered
			(d) If applicable, describe analytical methods taking account of sampling strategy	Covered
			(e) Describe any sensitivity analyses	Covered
Results	Participants	13*	(a) Report numbers of individuals at each stage of study. E.g., numbers potentially eligible, examined for eligibility, confirmed eligible, included in the study, completing follow-up, and analysed	Covered
			(b) Give reasons for non-participation at each stage	Covered
			(c) Consider the use of a flow diagram	Covered
	Descriptive data	14*	(a) Give characteristics of study participants (e.g., demographic, clinical, social) and information on exposures and potential confounders	Covered
			(b) Indicate the number of participants with missing data for each variable of interest	Covered
	Outcome data	15*	Report numbers of outcome events or summary measures	covered
	Main results	16	(a) Give unadjusted estimates and, if applicable, confounder-adjusted estimates and their precision (eg, 95% confidence interval). Make clear which confounders were adjusted for and why they were included	Covered
			(b) Report category boundaries when continuous variables were categorized	Covered
			(c) If relevant, consider translating estimates of relative risk into absolute risk for a meaningful time period	Not applicable
	Other analyses	17	Report other analyses done. E.g., analyses of subgroups and interactions, and sensitivity analyses	Covered
Discussion	Key results	18	Summarise key results with reference to study objectives	Covered
	Limitations	19	Discuss limitations of the study, taking into account sources of potential bias or imprecision. Discuss both the direction and magnitude of any potential bias	Covered
	Interpretation	20	Give a cautious overall interpretation of results, considering objectives, limitations, multiplicity of analyses, results from similar studies, and other relevant evidence	Covered
	Generalisability	21	Discuss the generalisability (external validity) of the study results	Covered
Other information	Funding	22	Give the source of funding and the role of the funders for the present study and, if applicable, for the original study on which the present article is based	Covered

Appendix B

Table 7 features the participant inclusion and exclusion criteria applied to the patients assessed consecutively (total n = 78) at the CNPP outpatient clinic in Kinshasa between April 1st and May 1st, 2025.

**Table 7:** Inclusion and exclusion criteria applied to the patients assessed

Criteria	No. of participants
Antipsychotic treatment <5 weeks	4
Age <18	1
Unable to complete questionnaires	3
Other	1
Declined participation/no consent	7
Enrolled and completed the assessment	62
Included in analysis	62

Appendix C

Table 8 presents the EqCPZ conversion ratios used in this study.

**Table 8:** EqCPZ conversion ratios For haloperidol decanoate, the administered dose and injection interval were first converted to an average daily oral equivalent dose, then converted to EqCPZ using the haloperidol oral ratio (2 mg = 100 mg EqCPZ). The EqCPZ was analysed per 100 mg increments. EqCPZ: Chlorpromazine equivalent (mg/day)

Antipsychotic	Dose = 100 mg EqCPZ	Reference
Chlorpromazine	100 mg	Atkins et al., 1997 [15]
Haloperidol (oral)	2 mg	Gardner et al., 2010 [16]
Risperidone	2 mg	Gardner et al., 2010 [16]
Olanzapine	5 mg	Leucht et al., 2015 [18]
Haloperidol decanoate (LAI)	Daily dose derived from injection interval	Inada & Inagaki, 2015 [17]

Appendix D

The VIF diagnostics are featured in Table 9.

**Table 9:** VIF diagnostics for logistic regression models A VIF <5 is generally considered acceptable. All VIF values are below 3, indicating no problematic multicollinearity. VIF: Variance inflation factors, EqCPZ: Chlorpromazine equivalents (mg/day), DAI: Drug Attitude Inventory, BIS: Birchwood Insight Scale, GASS: Glasgow Antipsychotic Side-effect Scale

Variable	VIF	VIF
	(Clinical model)	(Extended model)
Gender male	1.06	1.27
Accompaniment	1.24	1.37
Typical (reference atypical)	2.06	2.18
Combination (reference atypical)	2.75	2.94
Polypharmacy	1.70	1.75
EqCPZ/100	1.79	2.33
Halo decanoate	1.50	2.01
DAI	-	2.34
BIS	-	2.23
GASS	-	2.52

Appendix E

Tables 10-13 feature item-total statistics and internal consistency.

**Table 10:** MARS item-total statistics (Cronbach's alpha = 0.830, k=10) MARS: Medication Adherence Rating Scale, SD: Standard deviation

Item	Mean	SD	Corrected Item	Alpha of item
			Total r	deleted
MARS-01	0.532	0.503	0.566	0.809
MARS-02	0.484	0.504	0.521	0.814
MARS-03	0.581	0.497	0.399	0.826
MARS-04	0.710	0.458	0.707	0.796
MARS-05	0.839	0.371	0.541	0.814
MARS-06	0.452	0.502	0.345	0.832
MARS-07	0.661	0.477	0.673	0.799
MARS-08	0.597	0.495	0.444	0.822
MARS-09	0.532	0.503	0.646	0.801
MARS-10	0.500	0.504	0.395	0.827

**Table 11:** DAI item-total statistics (Cronbach's alpha = 0.758, k=10) DAI: Drug Attitude Inventory, SD: Standard deviation

Item	Mean	SD	Corrected item	Alpha of item
			Total r	deleted
DAI1	0.484	0.882	0.491	0.729
DAI2	0.258	0.974	0.465	0.732
DAI3	0.387	0.930	0.443	0.735
DAI4	0.097	1.003	0.472	0.730
DAI5	-0.032	1.008	0.368	0.746
DAI6	0.548	0.843	0.353	0.747
DAI7	0.290	0.965	0.469	0.731
DAI8	-0.032	1.008	0.339	0.750
DAI9	0.290	0.965	0.579	0.715
DAI10	0.226	0.982	0.245	0.763

**Table 12:** BIS item-total statistics (Cronbach's alpha = 0.730, k=8) The BIS item 2 has the lowest corrected item-total correlation (r=0.136). Removing it would raise alpha to 0.752. The item was retained for content validity and comparability. BIS: Birchwood Insight Scale, SD: Standard deviation

Item	Mean	SD	Corrected Item-	alpha if item
			Total r	deleted
BIS1	0.839	0.834	0.249	0.736
BIS2	0.387	0.754	0.136	0.752
BIS3	1.081	0.929	0.359	0.717
BIS4	1.323	0.719	0.596	0.672
BIS5	1.581	0.780	0.659	0.656
BIS6	1.323	0.883	0.663	0.648
BIS7	0.774	0.895	0.408	0.705
BIS8	1.145	0.865	0.372	0.713

**Table 13:** GASS item-total statistics (Cronbach's alpha = 0.963, k=23) The GASS item 21 (sexual function) has a negative corrected item-total correlation (r=-0.094). Removing it would raise alpha to 0.969. This item may reflect a culturally sensitive topic. GASS: Glasgow Antipsychotic Side-Effect Scale, SD: Standard deviation

Item	Mean	SD	Corrected item	Alpha of item
			Total r	deleted
GASS1	1.258	1.280	0.570	0.963
GASS2	0.919	1.219	0.772	0.961
GASS3	0.935	1.240	0.739	0.961
GASS4	0.823	1.153	0.789	0.961
GASS5	1.016	1.194	0.810	0.961
GASS6	0.710	1.151	0.874	0.960
GASS7	0.742	1.200	0.857	0.960
GASS8	0.790	1.282	0.811	0.960
GASS9	1.048	1.286	0.721	0.961
GASS10	0.742	1.200	0.819	0.960
GASS11	0.984	1.261	0.706	0.962
GASS12	1.065	1.329	0.623	0.963
GASS13	0.613	1.107	0.881	0.960
GASS14a	0.742	1.200	0.817	0.960
GASS14b	0.855	1.143	0.780	0.961
GASS15	0.855	1.185	0.767	0.961
GASS16	1.323	1.303	0.525	0.964
GASS17	0.710	1.165	0.885	0.960
GASS18	0.581	1.110	0.924	0.960
GASS19	0.742	1.186	0.841	0.960
GASS20	0.661	1.159	0.813	0.961
GASS21	0.581	1.195	-0.094	0.969
GASS22	1.935	1.447	0.432	0.965

Appendix F

The Firth's penalized (bias-reduced) logistic regression (Tables 14-16) was performed as a sensitivity analysis given sparse cells and possible quasi-complete separation for some predictors in the standard maximum likelihood estimation (MLE) models.

**Table 14:** Firth's univariable logistic regression for key psychometric predictors DAI: Drug Attitude Inventory, BIS: Birchwood Insight Scale, GASS: Glasgow Antipsychotic Side-effect Scale, CI: Confidence interval

Predictor	Firth's OR (95% CI)	Wald chi2	p-value
DAI (per 1-point)	0.65 (0.53-0.80)	16.78	0.000041952394060
BIS (per 1-point)	0.59 (0.45-0.77)	14.73	0.000124156476075
GASS (per 1-point)	1.10 (1.03-1.17)	8.10	0.004435648517012

**Table 15:** Firth's clinical/treatment model CI: Confidence interval

Factor	Firth's aOR (95% CI)	Wald chi2	p-value
Gender (male)	3.87 (1.06-14.13)	4.21	0.040192797273461
Accompaniment (yes)	2.48 (0.62-9.95)	1.65	0.199137235415591
Typical (ref Atypical)	2.23 (0.41-12.18)	0.85	0.355515648092959
Combination (ref Atypical)	4.77 (0.33-68.91)	1.31	0.251793460303540
Polypharmacy (>=2)	0.38 (0.05-2.71)	0.94	0.332195583113998
EqCPZ (per +100 mg)	1.31 (0.95-1.82)	2.73	0.098384087216147
Haloperidol decanoate	0.07 (0.01-0.72)	4.99	0.025520492455906

**Table 16:** Firth's extended model (clinical and psychometric) Firth-penalized estimates are modestly attenuated toward the null, but direction and inference are unchanged. The DAI and BIS remain independently associated with poor adherence; GASS loses significance (p=0.158) in the fully adjusted Firth model. DAI: Drug Attitude Inventory, BIS: Birchwood Insight Scale, GASS: Glasgow Antipsychotic Side-effect Scale, CI: Confidence interval, aOR: Adjusted odds ratio

Factor	Firth's aOR (95% CI)	Wald chi2	p-value
Gender (male)	2.40 (0.43-13.48)	0.99	0.319832220769585
Accompaniment (yes)	0.51 (0.07-3.87)	0.42	0.516016443747165
Typical (ref Atypical)	3.46 (0.36-33.30)	1.15	0.282611541416936
Combination (ref Atypical)	0.44 (0.01-23.91)	0.16	0.688515582684885
Polypharmacy (>=2)	0.54 (0.04-6.78)	0.22	0.635991456619369
EqCPZ (per +100 mg)	0.87 (0.56-1.34)	0.39	0.532111073744334
Haloperidol decanoate	1.02 (0.06-17.50)	0.00	0.989260833651720
DAI (per 1-point)	0.72 (0.55-0.95)	5.64	0.017598745907693
BIS (per 1-point)	0.59 (0.36-0.95)	4.75	0.029333537178048
GASS (per 1-point)	1.13 (0.95-1.34)	1.99	0.158075470956230

Appendix G

The descriptive statistics of individual GASS items by adherence status are listed in Table 17.

**Table 17:** GASS item-level comparisons by adherence status The Mann-Whitney U test (two-sided) was applied. Good adherence: MARS ≥7; Poor adherence: MARS <7; MARS: Medication Adherence Rating Scale

Item	Total	Good adherence	Poor adherence	U	p-value
	Mean ± SD	Mean ± SD	Mean ± SD		
GASS1	1.26 ± 1.28	0.85 ± 1.13	1.75 ± 1.29	298.0	0.007089
GASS2	0.92 ± 1.22	0.29 ± 0.58	1.68 ± 1.36	220.0	5.0123e-05
GASS3	0.94 ± 1.24	0.35 ± 0.77	1.64 ± 1.34	231.0	8.7365e-05
GASS4	0.82 ± 1.15	0.26 ± 0.62	1.50 ± 1.29	233.5	7.0538e-05
GASS5	1.02 ± 1.19	0.53 ± 0.79	1.61 ± 1.34	263.0	0.001109
GASS6	0.71 ± 1.15	0.32 ± 0.68	1.18 ± 1.42	334.0	0.013892
GASS7	0.74 ± 1.20	0.35 ± 0.77	1.21 ± 1.45	338.0	0.016692
GASS8	0.79 ± 1.28	0.09 ± 0.38	1.64 ± 1.47	217.0	4.1711e-06
GASS9	1.05 ± 1.29	0.35 ± 0.73	1.89 ± 1.31	182.5	3.8241e-06
GASS10	0.74 ± 1.20	0.24 ± 0.65	1.36 ± 1.42	284.0	8.5798e-04
GASS11	0.98 ± 1.26	0.47 ± 0.83	1.61 ± 1.42	269.5	0.001176
GASS12	1.06 ± 1.33	0.71 ± 1.12	1.50 ± 1.45	336.0	0.025947
GASS13	0.61 ± 1.11	0.18 ± 0.52	1.14 ± 1.38	308.0	0.002015
GASS14a	0.74 ± 1.20	0.09 ± 0.38	1.54 ± 1.37	203.0	2.1024e-06
GASS14b	0.85 ± 1.14	0.41 ± 0.78	1.39 ± 1.29	270.0	9.8051e-04
GASS15	0.85 ± 1.19	0.35 ± 0.77	1.46 ± 1.32	256.0	3.7601e-04
GASS16	1.32 ± 1.30	0.85 ± 1.21	1.89 ± 1.20	277.5	0.002766
GASS17	0.71 ± 1.16	0.21 ± 0.54	1.32 ± 1.42	279.5	6.5315e-04
GASS18	0.58 ± 1.11	0.09 ± 0.29	1.18 ± 1.42	296.0	7.0099e-04
GASS19	0.74 ± 1.19	0.29 ± 0.68	1.29 ± 1.44	306.0	0.003186
GASS20	0.66 ± 1.16	0.18 ± 0.52	1.25 ± 1.43	290.0	8.0611e-04
GASS21	0.58 ± 1.19	0.71 ± 1.29	0.43 ± 1.07	520.0	0.368619
GASS22	1.94 ± 1.45	1.50 ± 1.52	2.46 ± 1.17	323.0	0.009252
